# Editorial: Exercise intervention for prevention, management of and rehabilitation from COVID-19

**DOI:** 10.3389/fphys.2023.1293229

**Published:** 2023-09-26

**Authors:** Marcel Bonay, Osama Abdelkarim, Achraf Ammar

**Affiliations:** ^1^ Université Paris-Saclay, Université de Versailles St Quentin (UVSQ), INSERM END-ICAP, Versailles, France; ^2^ Service de Physiologie-Explorations Fonctionnelles bi-sites, Hôpitaux Ambroise Paré et Bicêtre, Assistance Publique-Hôpitaux de Paris, Boulogne, France; ^3^ Faculty of Physical Education, Assiut University, Assiut, Egypt; ^4^ Institute for Sport and Sport Science, Karlsruhe Institute for Technology, Karlsruhe, Germany; ^5^ Department of Training and Movement Science, Institute of Sport Science, Johannes Gutenberg-University Mainz, Mainz, Germany; ^6^ High Institute of Sport and Physical Education Sfax, University of Sfax, Sfax, Tunisia; ^7^ Research Laboratory, Molecular Bases of Human Pathology, LR19ES13, Faculty of Medicine of Sfax, University of Sfax, Sfax, Tunisia; ^8^ Interdisciplinary Laboratory in Neurosciences, Physiology and Psychology: Physical Activity, Health and Learning (LINP2), UFR STAPS (Faculty of Sport Sciences), Université Paris Lumières (UPL), Paris Nanterre University, Nanterre, France

**Keywords:** exercise, prevention, rehabilitation, infection, COVID-19

Caused by the severe acute respiratory syndrome coronavirus 2 (SARS-CoV-2), the coronavirus disease 2019 (COVID-19) pandemic has greatly stimulated health research with numerous benefits for patient care, ranging from the development of new vaccines to the identification of at-risk populations and the recognition of the value of physical exercise interventions as an effective method of prevention, management, and rehabilitation of patients. In this Research Topic “*Exercise intervention for Prevention, Management of and Rehabilitation from COVID-19*”, studies explore the effects of physical activity on COVID-19 illness and mortality, the impact of rehabilitation programs on different populations of COVID-19 patients, and characterize difficulties and needs of patients or athletes with disabilities during COVID-19 lockdown.

An increase in mortality among hospitalized COVID-19 patients with previous sedentary lifestyles has been shown in many studies. Conversely, the beneficial effects of physical activity on COVID-19 outcomes and disease severity have been suggested. Recently, a meta-analysis involving one million patients assessed the hospitalization, intensive care unit (ICU) admissions, and mortality rates of COVID-19 patients with a history of physical activity involvement before the beginning of the pandemic (Rahmati et al., 2022). Interestingly, the types of exercises were also studied. Resistance exercise and combined aerobic and muscle strength training were significantly associated with reductions in COVID-19 hospitalizations, and COVID-19 ICU admissions, respectively. The authors reported a positive association between endurance exercise and reduction in COVID-19 mortality. In this Research Topic, a Mendelian randomization study assessed the causal influence of light and moderate-to-vigorous physical activity on COVID-19 susceptibility, hospitalization and severity (Zhang et al.). No significant effects were found for moderate to vigorous physical activity on COVID-19 outcomes. However, light physical activity reduced the risk of COVID-19 hospitalization and severe complications. Another review and meta-analysis assessed the association between physical activity before COVID-19 and the severity of illness and mortality in COVID-19 patients (Sittichai et al.). Subgroup analysis showed that physical activity for ≥150 min/week at a moderate intensity or ≥75 min/week at a vigorous intensity reduced the risks of severity and mortality. Vigorous PA reduced mortality risk, whereas moderate to vigorous PA reduced the risks of severity and mortality. Although the heterogeneity in physical activity patterns and severity definition constitutes a limitation of these meta-analysis studies, engaging in regular physical activity was shown to decrease the severity and mortality of COVID-19 patients. Several studies suggest that exercise intervention improves the functional capacities and psychological health status of COVID-19 patients who were discharged from the hospital. Focusing on the management and rehabilitation of post-COVID-19 Tunisian patients, Toulgui et al. assessed the effect of 4-week cardiorespiratory rehabilitation program including aerobic cycle endurance, strength training, and educational sessions. The authors reported significant improvements in dyspnea, lung function, 6-min walk work, and resting heart rate and diastolic blood pressure in 14 moderate to severe COVID-19 patients (Toulgui et al.). Another clinical trial showed that an 8-week multi-professional intervention increased physical fitness, reduced biomarkers of inflammation, and improved lipid and glucose metabolism in overweight COVID-19 survivors from southern Brazil (Sordi et al.). In a critical scoping review of the literature, Puce et al. evaluated the effects of COVID-19 on athletes with disabilities and para-athletes. Interestingly, the authors highlighted the lack of follow-up studies in these populations and recommended more attention towards their needs. In a case report, Crisafulli et al. highlighted the importance of personalized adapted motor activity in a COVID-19 patient with critical illness polyneuropathy and myopathy. Numerous studies have reported the psychological effects of COVID-19 lockdown. AlMarzooqi et al. found an association between body image perception and demographic factors among physically active individuals during the COVID-19 lockdown in Saudi Arabia. In Malaysia, Washif et al. evaluated the extent of changes in training practices, recovery, mental health, and sleep patterns of athletes during the COVID-19 lockdown. Finally, Wedig et al. discussed the potential interest of blood flow restriction to decrease loss of muscle mass and strength during acute infection, and to mimic high-intensity exercise intervention during convalescence.

The studies published in this Research Topic report promising findings on the beneficial effects of regular physical activity and/or adopting exercise interventions on promoting functional capacities and psychological status in COVID-19 survivors while reducing the severity and mortality of COVID-19 patients. However, most of these studies agreed on the difficulty of adapting a rehabilitation program and finding the optimal levels of physical activity in a given individual.
